# Investigating antibiotics in the NICU and patient safety

**DOI:** 10.3389/fcimb.2025.1563940

**Published:** 2025-05-01

**Authors:** Shelley M. Lawrence, James L. Wynn, David W. Kimberlin, Joseph B. Cantey

**Affiliations:** ^1^ Division of Neonatology, Department of Pediatrics, College of Medicine, University of Utah, Salt Lake City, UT, United States; ^2^ Division of Neonatal-Perinatal Medicine, Department of Pediatrics, University of Florida, Gainesville, FL, United States; ^3^ Department of Pathology, Immunology, and Laboratory Medicine, College of Medicine, University of Florida, Gainesville, Gainesville, FL, United States; ^4^ Division of Pediatric Infectious Diseases, Department of Pediatrics, College of Medicine, University of Alabama, Birmingham, AL, United States; ^5^ Division of Neonatology, Division of Allergy, Immunology, and Infectious Diseases, Department of Pediatrics, University of Texas Health San Antonio, San Antonio, TX, United States

**Keywords:** neonatal infection, antibiotic utilization, parental consent, Redbook guidelines, antimicrobial stewardship, patient safety - standards, study design and best practice

## Abstract

In recent years, a number of observational studies have been published in neonatology-focused journals that modify American Academy of Pediatrics (AAP) evidence-based guidelines, as outlined in the Red Book and other publications authored by the AAP Committee on the Fetus and Newborn and the AAP Committee on Infectious Diseases, for the treatment of neonatal infectious diseases. Specifically, studies that decrease the treatment length or advocate for the early transition of intravenous to oral antibiotics in all neonates with suspected or culture-positive sepsis or pneumonia should be regarded with caution. These studies are usually conducted through quality improvement initiatives without informed parental consent, and lack well-defined definitions for the infection of interest. They often fail to include sufficient information regarding well-documented variations in gestational age-based immune responses, and usually combine bacterial infections into a single group without considering virulence and pathophysiologic differences between gram-negative and gram-positive infections. We seek to raise awareness of the potential harm these practices may cause and to advocate for parental involvement and consent when deviations from practice standards are investigated.

## Introduction

Caution is necessary when considering practice changes that have not been rigorously studied to ensure patient safety. Recent publications promoting shortened empiric antibiotic courses for neonatal early-onset sepsis (EOS) (Sánchez et al., 2023) and blood culture-negative pneumonia (Lewald et al., 2023), or the prompt transition of intravenous antibiotics to oral agents (within 48–72 hours) for confirmed bacteremia or other serious bacterial infections (Keij et al., 2022; Malchau Carlsen et al., 2023) should warrant pause. These findings are largely based on observational data rather than clinical trials, and reports of parental consent/awareness that their infant was to receive a change in care that deviated from major academic society recommendations are not usually included. However, modification of evidence-based antibiotic treatment guidelines may heighten an infant’s risk of a poor outcome, including death, particularly for those requiring intensive care (Lewald et al., 2023; Sánchez et al., 2023). The lack of consensus definitions for neonatal sepsis and pneumonia causes doubt regarding the certainty of the diagnosis, as transient tachypnea of the newborn or respiratory distress syndrome in many preterm infants will improve over the course of the first few days of age and may be misconstrued as appropriately treated pneumonia or sepsis. Moreover, the Neonatal Research Network reported that nearly 20% of well-appearing but bacteremic term infants received care in the normal newborn nursery in the first 72 hours but had cultures primarily sent for maternal risk factors (Stoll et al., 2011). This finding signifies that bacteremia is not universally associated with sepsis, and infants can have serious bacterial infections without a positive blood culture result.

## Neonatal infections

Bacterial sepsis is the seventh leading cause of neonatal mortality in the United States. It is primarily surpassed by complications associated with premature birth, congenital malformations and genetic disorders, and high-risk maternal conditions (Centers for Disease Control and Prevention, 2017). In 2019, the Centers for Disease Control and Prevention recorded 603 neonatal deaths attributable to bacterial sepsis of the newborn, a 5.2% increase from the previous year (Centers for Disease Control and Prevention, 2021). While the overall incidence of early-onset sepsis (EOS or infection ≤ 72 hours of age) remains relatively stable at approximately 1.08 [95% confidence interval (CI), 0.95–1.23] cases per 1000 live births (LBs) (Stoll et al., 2020), the incidence of late-onset sepsis (LOS or infection > 72 hours of age) has declined over the past two decades from approximately 211 cases per 1,000 LBs to 88.5 (95% CI, 86.4–90.7) cases per 1,000 LBs in very low birth weight (VLBW; ≤1500 g) infants (Flannery et al., 2022). Significantly, rates of infection are inversely related to gestational age in the neonatal intensive care unit (NICU), with a 14-fold increase in EOS for VLBW infants compared with term infants (Stoll et al., 2020) and a 15.5-fold increase in LOS for infants born at ≤ 23 weeks of gestation compared with those born at > 29 weeks (Flannery et al., 2022). Bacteremia-associated morbidities and mortality also disproportionately impact infants at younger gestational ages. Case fatality rates from EOS were recorded in 38 out of 131 infants between 22 and 36 weeks of gestation, with no fatalities reported among those born at term (Stoll et al., 2020). Similarly, survival rates for LOS range from 62.9% at ≤ 23 weeks to 85.4% for those over 29 weeks of gestation (Flannery et al., 2022). Survivors of sepsis are more likely than age-matched controls without sepsis to require home oxygen, tracheostomy, and gastrostomy at any gestational age (Flannery et al., 2022).

Because common neonatal conditions have overlapping clinical and diagnostic findings, especially in preterm infants, clinicians must remain vigilant in their awareness of the risk of infection and maintain a low threshold for initiating antimicrobials. Many etiologies of bacterial infection in this population, including pneumonia, meningitis, necrotizing enterocolitis, urinary tract infection, osteomyelitis, and omphalitis, are not ubiquitously associated with a positive blood culture, and discontinuing antibiotics at 36–48 hours following sterile cultures would be inappropriate in patients with these infections. Sterile cultures in neonates exposed to intrapartum antibiotics for maternal chorioamnionitis or ‘Triple I’ (i.e., intrauterine inflammation, infection, or both) should also raise suspicion for partially treated bacterial infection in the newborn. Intrauterine infection remains a leading cause of spontaneous preterm birth, and neonatal blood cultures can be falsely negative due to low bacterial burden, limited sample volume, and prolonged administration of maternal antibiotics, as these drugs inhibit bacterial growth in culture (Goldenberg et al., 2008). Adequate culture volume (>1 mL) is critical in such cases to ensure adequate sensitivity to detect bloodstream infection (Schelonka et al., 1996).

## Discussion

The Red Book^®^, authored by the AAP and the Committee on Infectious Diseases, provides evidence-based recommendations regarding the medical management of neonates with infectious diseases. Publications authored by the Committee on Fetus and Newborn and the Committee on Infectious Diseases provide additional management guidance (Puopolo et al., 2018a; Puopolo et al., 2018b). Willful modification of these recommendations should be undertaken judiciously but may be appropriate in certain clinical situations. Because the overall incidence of neonatal bacteremia is low, evidence from expertly designed, multi-center quality improvement studies may prove helpful. However, these studies should consider differences in the incidence of infectious diseases based on gestational age, use consensus definitions for the infectious condition being studied (i.e., sepsis, pneumonia, other site-specific infections with sterile blood cultures, etc.), and obtain informed parental consent for study participation. Quality improvement approaches should utilize safety-monitoring committees, as they often attain institutional review board approval with a waiver of parental consent. Importantly, it is our belief that results from studies in adults or even older children of shortened courses of antibiotic treatment for specific conditions should not be extrapolated *carte blanche* to the premature neonate population. Just as children are not small adults, premature babies are not simply small children, nor are they the equivalent of a term infant. Preterm infants have substantial differences in innate and adaptive immune responses that not only place them at significant risk for infectious diseases but also for associated adverse long-term neurodevelopmental impairments (Mukhopadhyay et al., 2021).

In 1996, Drs. McGowan and Gerding (1996) introduced the term ‘antibiotic stewardship,’ with considerations for antimicrobial selection, dosage, and duration of treatment to address the rise in bacterial resistance. Since then, a surge in antibiotic stewardship initiatives in NICUs across the United States has led to significant decreases in antibiotic exposure. Antibiotic stewardship programs can improve patient safety and increase the frequency of correct antibiotic prescribing for therapy and prophylaxis while optimizing the treatment of infections and reducing adverse events associated with antibiotic use. Implementing the Kaiser Permanente Early-Onset Sepsis Calculator for late preterm and term infants decreased empiric antibiotics for EOS by nearly half in the first 24 hours of life without significant adverse effects (Kuzniewicz et al., 2017). Hard stops or antibiotic alerts at 36–48 hours following a sepsis evaluation with sterile blood cultures and a well-appearing infant have also reduced the unnecessary use of antimicrobials by nearly 28% (Astorga et al., 2019). However, even fierce advocates for antibiotic stewardship acknowledge that stewardship efforts should not include withholding antibiotics from neonates demonstrating clinical signs of infection or truncating current practice standards of the duration of antibiotic treatment in those with suspected or culture-confirmed bacterial infections. Such practices may heighten the risk of poor outcomes, including death, for neonates requiring intensive care (Singh et al., 2024). For neonatal antibiotic stewardship, the goal is not to drive antibiotic usage inexorably toward zero but rather to make sure that antibiotics are used correctly.

Both over- and under-utilization of any medication is associated with risks. The limited sensitivity of available sepsis screening tools and the absence of defined criteria to study neonatal infectious diseases do not support withholding antimicrobial treatment in neonates with suspected infection or shortening courses of therapy when clinical judgment is highly suspicious for a sepsis episode, even when the laboratory cultures remain negative. Close clinical monitoring of these infants is also crucial when antimicrobial agents are discontinued. While investigations regarding the appropriate length of exposure to antimicrobial agents should be undertaken, patient safety must remain our top priority.

## Data Availability

The original contributions presented in the study are included in the article/supplementary material. Further inquiries can be directed to the corresponding author.
